# A Bibliographic Assessment Using the Degrees of Publication Method: Medicinal Plants from the Rural Greater Mpigi Region (Uganda)

**DOI:** 10.1155/2021/6661565

**Published:** 2021-01-15

**Authors:** Fabien Schultz, Godwin Anywar, Cassandra Leah Quave, Leif-Alexander Garbe

**Affiliations:** ^1^Institute of Biotechnology, Faculty III - Process Sciences, Technical University of Berlin, Gustav-Meyer-Allee 25, Berlin 13355, Germany; ^2^Department of Agriculture and Food Sciences, Neubrandenburg University of Applied Sciences, Brodaer Str. 2, Neubrandenburg 17033, Germany; ^3^Department of Dermatology, Emory University School of Medicine, 615 Michael St., Atlanta, 30322 GA, USA; ^4^Department of Plant Sciences, Microbiology and Biotechnology, Makerere University, P.O Box 7062, Kampala, Uganda; ^5^Center for Study of Human Health, Emory University College of Arts and Sciences, 615 Michael St., Atlanta, 30322 GA, USA; ^6^ZELT - Neubrandenburg Center for Nutrition and Food Technology gGmbH, Seestraße 7A, Neubrandenburg 17033, Germany

## Abstract

In ethnopharmacological research, many field assessment tools exist. Yet, these miss that critical point of how to really determine which species merit the costly lab studies, e.g., evaluation of traditional use via pharmacological assays and isolation of bioactive secondary metabolites. This gap can be filled with the introduction of a new tool for literature assessment: the Degrees of Publication (DoPs). In this study, its application is illustrated through an extensive bibliographic assessment of 16 medicinal plant species that were recently identified in the Greater Mpigi region of Uganda as being frequently used by local traditional healers in the treatment of medical disorders (namely, *Albizia coriaria*, *Cassine buchananii*, *Combretum molle*, *Erythrina abyssinica*, *Ficus saussureana*, *Harungana madagascariensis*, *Leucas calostachys*, *Microgramma lycopodioides*, *Morella kandtiana*, *Plectranthus hadiensis*, *Securidaca longipedunculata*, *Sesamum calycinum* subsp. *angustifolium*, *Solanum aculeastrum*, *Toddalia asiatica*, *Warburgia ugandensis,* and *Zanthoxylum chalybeum*). These species are suspected to be understudied, and a thorough bibliographic assessment has not been previously performed. Thus, the objectives of our study were to undertake a comparative assessment of the degree to which each of these plant species has been studied in the past, including evaluation of the quality of the journals where results were published in. The determination of the DoPs enabled successful assessment of the degrees to which each individual plant species has been studied so far, while also taking into account the methodological “research chain of ethnopharmacology” from ethnobotanical studies (“traditional use”) to pharmacological assays (“bioactivity”) and finally to pharmacognostic research (“structure elucidation”). The significance of a research paper was assessed by determining whether its journal and publishing house were members of the Committee on Publication Ethics (COPE). In total, 634 peer-reviewed publications were reviewed covering the period of 1960–2019, 53.3% of which were published in journals and by publishing houses affiliated with COPE (338 publications). The literature assessment resulted in the identification of understudied plants among the selected species. The majority of plants reviewed have not been sufficiently studied; six species were classified as being highly understudied and three more as being understudied: *C. buchananii*, *F. saussureana*, *L. calostachys*, *M. lycopodioides*, *M. kandtiana*, and *S. calycinum* subsp. *angustifolium* and *A. coriaria*, *P. hadiensis,* and *S. aculeastrum,* respectively. The newly introduced DoPs are a useful tool for the selection of traditionally used species for future laboratory studies, especially for pharmacological bioassays, isolation procedures, and drug discovery strategies.

## 1. Introduction

Throughout human history and across the globe, plants were regarded as the major source of medicine and natural remedies. Traditional medicine is defined by the World Health Organization (WHO) as “the knowledge, skills, and practices based on the theories, beliefs, and experiences indigenous to different cultures, used in the maintenance of health and in the prevention, diagnosis, and improvement or treatment of physical and mental illness” [1]. In the developing world, over 80% of the population still rely on traditional herbal medicines for their day-to-day healthcare needs [2–4]. This is largely attributed to their ease of access, affordability, perceived fewer side effects, and cultural appropriateness, among other reasons [5]. Despite the general loss of cultural practices worldwide [6, 7], traditional medicine practices and medicinal plant use are still the predominant form of healthcare services in East and Central Africa today [8, 9]. The global importance of plants as a source of medicine is also often emphasized by scientists worldwide [10–14]. Around 25% of the Western drugs prescribed contain active ingredients that were initially isolated as natural products from plants [10]. Still, the majority of Earth's plant species has never been screened for pharmacological effects in a research facility [10, 15].

In consideration of this global importance, there are many assessment tools applied when reporting field studies in the science of ethnopharmacology. These include field assessment indices for medicinally used species, such as the frequency of citation, use value, informant consensus factor, and fidelity level, among others. However, none of these take into account how to really determine which species merit the costly lab studies. This is why we introduce the Degrees of Publication (DoPs), providing a standardized way to examine how well studied individual species are (or are not) in an ethnopharmacological context. In this study, 16 medicinal plant species from the Greater Mpigi region were selected to illustrate how the new tool works.

Situated in West-Central Uganda, the tropical Greater Mpigi region displays a high abundance of traditional medicine practitioners and diverse use of a vast amount of medicinal plant species [14, 16, 17]. Consequently, local people are still highly dependent on these traditional healers and their medicinal plants in order to secure their primary health care.

A recently published ethnobotanical survey from the Greater Mpigi region [14] and an ethnopharmacological study [18] identified 16 medicinal plant species that are often used in the treatment of medical disorders in the local traditional medicine system while displaying high pharmacological activity in our ongoing *in vitro* evaluation in a lab setting. A preliminary literature review resulted in a few results. Therefore, these 16 plants are suspected to be understudied species, and a thorough literature review using the new DoP method for bibliographic assessment enables the selection of traditionally used species for pharmacological bioassays and drug discovery strategies. Our study, therefore, aims to undertake a comparative literature assessment, applying the DoP method, regarding (a) other reports of these species, (b) the quality of the journals where results were published in (assessment of international standards and best practice in scholarly publication ethics), and (c) the degree to which each plant species has been studied thus far.

## 2. Materials and Methods

### 2.1. Study Objects

Our study objects are 16 tropical plant species identified to be frequently used by Ugandan traditional healers in treatment of diverse medical disorders in the Greater Mpigi region. This choice of species can be considered taxonomically diverse, representing 13 different plant families.  Table 1 lists these species, stating their scientific names, local names at the study site (Luganda language), their plant families, and their Relative Frequencies of Citation (RFC), calculated from absolute values of the ethnobotanical survey (*n* = 39) previously published by Schultz et al. [14].

### 2.2. Literature Review

Our research strategy included prioritization of some of the collected plant species for future pharmacological bioassays. Here, the results of the ethnobotanical survey on traditional use were the major indicator [14]. However, another parameter for this assessment was conducting a literature survey, identifying those medicinal plant species that are currently understudied, hereby limiting duplication of research efforts.

A literature search of electronic databases included GoogleScholar and the Web of Science Core Collection, using the scientific name of each plant as keywords (synonyms included). As suggested by Heinrich et al. [19], NAPRALERT^®^, a comprehensive natural-product database containing ethnomedical and pharmacological information of extracts and isolated compounds, was consulted as an additional tool (http://www.napralert.org). Membership in the Committee on Publication Ethics (COPE) was assessed by searching for an individual journal and the corresponding publishing house on the COPE website (http://www.publicationethics.org/members). Digitalized herbarium voucher specimens were obtained from the JSTOR Global Plants Database (https://plants.jstor.org).

### 2.3. Data Analysis: Degrees of Publication

The results of the literature survey were analyzed by categorizing published studies on the 16 medicinal plant species. A new indicator was introduced: the Degrees of Publication (DoPs). DoPs were defined as “Traditional Use,” “Bioactivity,” “Structure Elucidation,” “Other,” and “Total (without <other>).” “Traditional use” are sources stating that a plant species *i* is used traditionally in an ethnopharmacological context. The DoP “Bioactivity” describes the number of published studies investigating a potential pharmacological activity of an extract from a plant *i*. “Structure elucidation” includes studies that resulted in isolation of (bioactive) secondary metabolites and their structure elucidation. These three DoPs are consecutive steps in the bioassay-guided discovery of novel bioactive natural products based on the ethnopharmacological approach of investigating traditional use reports. This classification, therefore, may lead to an assessment of the degree to which each plant species has been studied so far. The DoP “Other” classifies all publications mentioning a plant species *i* in a non-ethnopharmacological context (e.g., studies on the morphology of the species, on the distribution of species, or on non-medicinal traditional use of a species). The DoP “Total (without <other>)” summarizes the three ethnopharmacologically relevant DoPs and is defined as the sum of “Traditional Use,” “Bioactivity,” and “Structure Elucidation.” For each DoP, absolute numbers were given as value *N*_*all*_ and *N*_*COPE*_, whereas *N*_*all*_ describes all publications discovered in the literature survey, and *N*_*COPE*_ lists all publications in scientific journals whose publishing houses are members of the Committee on Publication Ethics (COPE, http://www.publicationethics.org). Members of the COPE must accept the international standards and best practice in the ethics of scholarly publishing, meaning that membership of the COPE is an appropriate indicator for high-quality research. A DoP “Total (without <other>)” of *N*_*all*_ ranging from 0 to 14 classifies a plant species as being “highly understudied,” while 15–29 is “understudied,” 30–44 is “moderately studied,” and 45-∞ is “highly studied.”

## 3. Results and Discussion

### 3.1. Species Information

**Figure 1:**
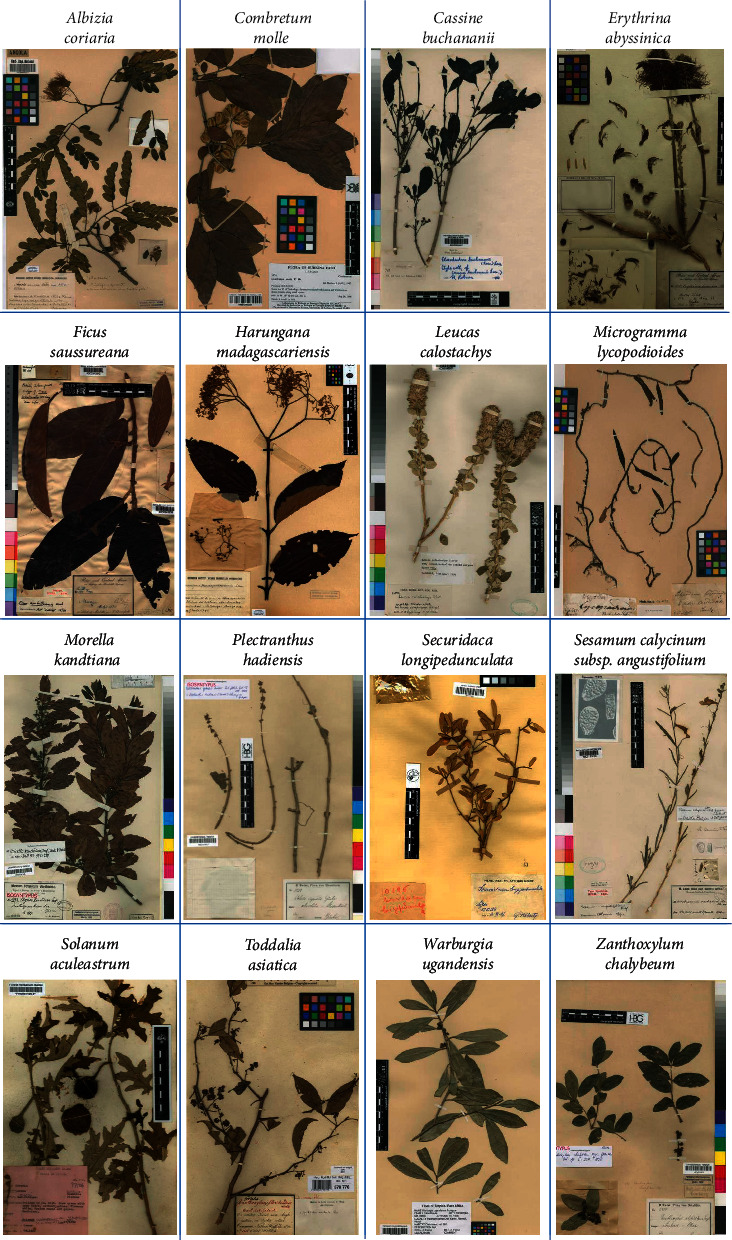
Digitized herbarium voucher specimens showing the 16 selected medicinal plant species used in the Greater Mpigi region, Uganda (source: JSTOR Global Plants Database).

Figure 1 is a compilation of digitized herbarium voucher specimens to give an overview of the appearance of each of the 16 plant species. Sections 3.1.1–3.1.16 provide information on synonyms, geographical distribution (in Uganda, in particular), life forms, ecological growth conditions and climate zones, local names in East Africa, and some basic characteristics for each of the selected plants.

#### 3.1.1. *Albizia coriaria*

*A. coriaria* is a pioneer tree that is found throughout Uganda on forest edges, wooded grasslands, woodland, and thickets. The tree is large and deciduous. Although it can reach a height of up to 18 m, it is frequently smaller with a flat, spreading crown [20, 21]. It is an indigenous plant that is also known as the “giant albizia” [20, 22]. *A. coriaria* can generally be found from Sudan to southern Angola [20]. It grows on various soil types at an altitude of 850–1,680 m above sea level (m.a.s.l.) [23]. *A. coriaria* can be propagated from seeds, and wild plants can be collected and planted. The seeds have a good germination rate [23]. The stem bark was formerly utilized as a fish poison in the Madi and West Nile areas of Uganda [24]. Local names in different Ugandan languages are as follows: **Luganda:** mugavu [14, 20–23], **Lusoga:** musita [20, 21, 23], **Ateso:** etek and etekwa [20, 23], **Kwamba:** musisiya [23], **Lugishu:** chesovio and kumoluko [23], **Swahili:** mugavu [20, 22], **Lugwe:** mubere [23], **Luo (Acholi):** latoligo and ayekayek [20, 23], **Luo (Jopadhola):** omogi and ober [20, 23], **Luo (Langi):** itek and bata [23], **Madi:** oyo [23], **Rukiga:** muyenzayenze [23], **Runyankore:** musisa and murongo [20, 23], **Runyoro:** musisa [20, 23], **Rutoro:** musisa [20, 23], and **Ik:** kiluku [21]. A local name in other East African countries is as follows: **Luhya**: omubele [20].

#### 3.1.2. *Cassine buchananii*

Some synonyms include: *Elaeodendron buchananii* (Loes.) Loes., *E. keniense* Loes., *E. stolzii* Loes., *E. warneckei* Loes., *E. afzelii* Loes., and *E. friesianum* Loes. This indigenous species is better known as the “moth tree” or the “leathery-leaved saffron” [20]. It is a small shrub to large tree up to 24 m high with a round compact crown that commonly occurs in grasslands in parts of Uganda [24, 25], but can also be found in dry upland evergreen forests, forest remnants, and riverine woodland (growing at an altitude of 1,200–2,100 m.a.s.l.) [20]. Its ripe fruits are green-orange and ovoid (up to 2.5 cm). Parts of the tree are known to be extremely toxic to livestock, especially when the leaves are ingested. Death occurs suddenly. Interestingly, giraffes eat the leaves of *C. buchananii* without notable adverse effects [24]. The local name in Uganda is as follows: **Luganda**: mbaluka [14, 21, 26]. Local names in other East African countries are as follows: **Kisii:** enkanda [24], **Meru:** mutimweru [24]; **Kikamba:** mutanga and mutanya [24], **Kipsigis/Lumbwa:** sawanet [24], **Sebei:** sunwa [24], and **Kinyaramba:** mtuwilang'holo [24].

#### 3.1.3. *Combretum molle*

Some of the synonyms are *C. welwitschii* Engl. & Diels, *C. arbuscula* Engl. & Gilg, *C. nyikae* Engl., *C. boehmii* Engl., *C. holtzii* Diels, *C. schelei* Engl., and *C. ankolense* Bagsh. *C. molle* is a slow-growing tree widespread in wooded grasslands and bushlands in Uganda and the rest of the African continent. It also commonly grows on stony hills up to an altitude of 2,300 m.a.s.l. [20]. The seeds germinate easily if fresh [23]. It is usually 5–7 m in height and branching near its base [20]. The names in Ugandan local languages are as follows: **Ateso:** ekworo and eworo [21, 23], **English:** velvet-leaved *Combretum* and velvet bushwollow [20, 23], **Luganda:** ndagi [14, 20, 21, 23], **Lugbara:** geleo [23], **Lugishu:** shikimira [23], **Lugwe:** muchuta [23], **Lugwere:** kinakworo [23], **Luo (Acholo):** okechu and oduk [20, 23], **Luo (Jopadhola):** deda [23], **Luo (Langi):** iworo and iyoro [23], **Lusoga:** ndawa, daha, and nfodwa [23], **Madi:** otubi and lebilebi [23], **Runyoro:** murama [23], **Sebei:** kembei [23], and **Ik** : ngulara [21]. Local names in other regions of East Africa are as follows: **Luhya**: mukhungula [20], **Maasai:** ol-mororoi [20], **Swahili:** mgurure [20], **Sukuma:** kagua [20], **Kamba:** muama [20], **Kikuyu/Meru:** murema and murama [20], **Taita:** mwama [20], and **Haya/Nyamwezi:** mlama [20].

#### 3.1.4. *Erythrina abyssinica*

*E. abyssinica* is a deciduous tree, reaching a height of 6–12 m. It has a short trunk and thick spreading branches. It has a rounded crown and occurs in savannah woodland, grassland, and scrubland throughout Uganda [20, 22, 23]. It propagates through seeds and cuttings, but the seeds have a low germination rate. *E. abyssinica* is an indigenous species that is also known as the “red-hot poker tree,” the “flame tree,” the “Uganda coral tree,” or the “lucky bean tree” [20, 22]. The tree is called “flame tree” because of its orange-red flowers. Common synonyms are *E. bequaerti* De Wild., *E. kassneri* Baker f., *E. tomentosa* R. Br., *Chirocalyx abyssinicus* (Lam.) Hochst., *C. tomentosus* Hochst., and *Corallodendron suberifera* (Welw. ex Baker) Kuntze. The local names in different languages in Uganda are as follows: **Luganda:** muyirikiti and jirikiti [14, 20–23], **Lugbara:** oluo and olugo [21, 22], **Runyankore:** muko, kiko, and murinzi [14, 23], **Lugishu:** cheroguru and muragolo [20, 22, 23], **Lugwe:** mutembetembe [22, 23], **Lunyuli:** mudongodongo and mukobe [22, 23], **Swahili:** mwamba-ngoma [20, 22], **Luo (Acholi):** lochoro, kisoro, oding, and loting [22, 23], **Luo (Jophadhola):** koli [23], **Luo (Langi):** ewilakot [23], **Madi:** olawu [22, 23], **Rukiga:** bwiko [23], **Runyoro:** mudoti, muko, and kiko [23], **Rutoro:** muko and kiko, **Sebei:** kaborte [23], **Ateso:** engosorot [23], **Kwamba:** kikiri [23], and **Lusoga:** muyirikiti [21]. Local names in other East African countries include the following: **Chagga:** mriri [20, 22], **Kamba:** muvuti [20], **Taita:** mulungu [20, 22], **Kisii:** omotembe [20, 22], **Hehe:** muhemi [20, 22], **Pare:** muungu [20, 22], and **Ateso:** engosorot [20, 22].

#### 3.1.5. *Ficus saussureana*

Some synonyms include *F. eriobotryoides* Kunth & C.D. Bouché, *F. afeelii* Kunth and C.D.Bouché, *F.dawei* Hutch, *F. murrayana* Miq., *F*. *monbuttuensis* Warb., *F. dawei* Hutch, or *Galoglychia saussureana* Gasp. It is a large, mostly epiphytic, hemi-epiphytic, or terrestrial tree [27, 28]. The base of the trunk consists of a mass of fused aerial roots. It produces large amounts of white latex. The slash typically discolours, but the latex does not [28]. It is a widely distributed tree in West Africa and the eastern and western margins of the Congo Basin [29]. In Uganda, it mainly occurs in the northern, western, and south-central parts [28]. *F. saussureana* prefers riverine, groundwater, and lowland forest areas [27]. The local name in **Luganda** language is as follows: muwo [14, 21].

#### 3.1.6. *Harungana madagascariensis*

Some common synonyms include: *Haronga madagascariensis* (Lam. ex Poir.), *Haronga paniculata* Lodd. ex Steud., *Haronga pubescens* Steud., and *Arungana paniculata* Pers. The vernacular name is “orange-milk tree” [20]. It is a pioneer, evergreen shrub or tree, reaching 3–18 m in height, whose bark, leaves, and stem produce a brilliant orange sap that turns blood-red on exposure. The outer layers of the wood and the innermost layer of the bark yield a yellow sap. This sap is traditionally used as a dye [20, 23, 30]. The bark mixed with the highly poisonous *Mansonia altissima* is used as Bété arrow poison in the Daloa region of the Ivory Coast [30]. *H. madagascariensis* occurs throughout tropical Africa, from Senegal to East Africa. It is a common and widely distributed pioneer tree species in Uganda, where it grows along forest edges, in areas where forests have been cleared, in secondary scrubland, around termite mounds, and in riverine areas at medium to low altitudes [20, 23, 30]. The local names in different languages in Uganda are as follows: **Luganda**: mulirira and mukabiiransiko [14, 20, 23, 30], **Madi:** asonbere and serubele [14, 30], **Rukiga:** mungolero, munianga, and muliamanga [23], **Runyankore**: mutaha [20, 23], **Rutoro**: murinda, murunda, and musoga [20, 23, 30], **Luo:** aremo [20], **Kirundi:** umushayishyi [30], **Nyankole:** omutaha [30], **Kiga:** omungolero mniananga and muliamanga, and **Swahili:** mkekundu, mdamudamu, mpulapula, nkekundu, nrimba, ngoningoni, kunamaji, funa maji, mdura, and mgondogado [30]. Local names in other East African languages and countries are as follows: **Luhya-Bukusu:** namalasile [20], **Luhya-Kisa:** omwinyala amatsai [20], **Nandi:** chepsebil [20], **Meru:** munyanwe [20], **Embu:** munyanwe [20], **Sambaa:** mkuntu [20], **Ngindo:** muhekara [30], **Mbunga:** mtelekajugo [30], **Rufiji:** mulungamo [30], **Pogoro:** msongoliko [30], **Hehe:** mtunu [30], and **Digo:** marindazia [30].

#### 3.1.7. *Leucas calostachys*

Synonyms are *Leucas calostachys* var. calostachys and *Leucas calostachys* var. *fasciculata* (Baker) Sebald. *L. calostachys* is an aromatic herb that occurs in some parts of Uganda, including the Greater Mpigi region [14]. However, there is limited literature on this species. The local name in **Luganda** language is as follows: kakuba musulo [14].

#### 3.1.8. *Microgramma lycopodioides*

Known synonyms for this species are *Pleopeltis lycopodioides* (L.) C. Presl, *Polypodium lycopodioides* L., *Niphobolus lycopodioides* (L.) Keyserl., and *Phymatodes lycopodioides* (L.) Millsp. It is an epiphytic or terrestrial fern that has been reported in tropical America, especially Brazil and Mexico, in sub-Saharan Africa, and in the Caribbean [31–38]. In Uganda, *M. lycopodioides* has been recorded in Masaka district, Lake Nabugabo, Mengo, Entebbe, Kibale forest, and in the Greater Mpigi region [14, 39, 40]. The local name in Uganda is as follows: **Luganda**: kukumba [14].

#### 3.1.9. *Morella kandtiana*

There is one synonym: *Myrica kandtiana* Engl. *M. kandtiana* is an herb, shrub, or short multibranched tree that spreads. The flowers are on the inflorescences, which are greenish yellow. The inflorescences occur on the older rather than on the younger branches. It grows in grasslands, in seasonal swamps, or swampy areas, but is very rare nowadays [41, 42]. Local names in different languages in Uganda are as follows: **Luganda:** mukikimbo, bowolola omusajja, and enkikimbo [14, 41, 43] and **Runyankore/Runyoro:** omujeje [41].

#### 3.1.10. *Plectranthus hadiensis*

Common synonyms are *P. cyaneus* Gürke, *P. forsskaolii* Vahl, *Coleus personatus* Lem., and *C. forsskaolii* Briq. It is a widespread, semi-succulent, herbaceous perennial herb in East and Central Africa. It has also been reported in South Africa. *P. hadiensis* can grow 10–150 cm high [41, 44, 45]. Local names in Uganda are as follows: **Luganda:** mukikimbo [14] and **Lusoga:** kiraga and kigalama [41].

#### 3.1.11. *Securidaca longipedunculata*

Some of the common synonyms include *Elsota longipedunculata* (Fresen.) Kuntze and *S. longipedunculata* var. *longipedunculata*. It is a semi-deciduous shrub or small tree that can reach a height of 2–6 m. *S. longipedunculata* is widespread throughout tropical Africa from Kenya and Uganda to South Africa. It occurs in wooded and savannah grassland and woodland, preferring dry areas, and it is associated with *Hymenocardia acida* and *Combretum* spp. The plant easily propagates through seedlings, but seeds germinate with difficulty if not pretreated. The roots are yellow, and if cut, this species radiates an intense aromatic smell. The flowers are sweet scented, in numerous racemes, and magenta, purple, or violet in color [23, 24, 30]. According to Neuwinger [30], *S. longipedunculata* is “one of the most beautiful African flowering shrubs or trees.” Interestingly, the plant is highly toxic to humans, which is why it has been used as a hunting poison in Africa, but much more often as a trial-by-ordeal and murder poison. For example, the plant has been described as the most often used ordeal poison among the Gbaya people in the Central African Republic. Sadly, the Lunda women of the Democratic Republic of Congo, Zambia, and Angola consider the root pulp or the peeled root the “best known and most effective of all the intravaginal poisons” used for suicide [30]. Local names in different languages in Uganda are as follows: **Ateso:** elilyoi and elilie [23], **Lugbara:** oiyofe [23], **Lugishu:** wadambasima [23], **Lugwe:** mwiabala and amwiabala [23, 30], **Lugwere:** loloyi [23, 30], **Luo (Acholi):** aliya, lalia, and lalon [23, 30], **Luo (Jophadhola):** lilyo [23, 30], **Luo (Langi):** elila [23, 30], **Madi:** lio [23, 30], **Runyankore:** mweya and omweya [23, 30], **Runyoro:** nkondwe and nkungwe [23, 30], **Luganda:** lilo and mukondwe [14, 21, 23, 30], **Swahili:** Nzigi, muteya, matungunungu, and mzigi [30], **Lusoga:** mukondwa [21, 23], **Teso:** elilyoi and elilie [30], and **Soga:** mukondwa [30]. Local names in other East African countries and languages are as follows: **Nyarwanda:** umunyagazozi and umukuyu [30], **Kirundi:** umunyagazozi [30], **Hehe:** muhulatangu and mukenegatangu [30], **Zigua:** mkola and mkala [30], **Zinza:** mweyo [30], **Sukuma:** hengo-hengo, nengo-nengo, and mbaso [30], **Yao:** chiguluka [30], **Ngindo:** kiguraka [30], **Mwera:** mtikwi [30], **Shambaa:** mbazo [30], **Kamba:** ithithi [30], **Kikuyu:** muguraka [30], and **Digo:** muteya, mzidvi, mzidyi, and mzisi [30].

#### 3.1.12. *Sesamum calycinum* Subsp. *angustifolium*

There are two synonyms: *Sesamum angustifolium* (Oliv.) Engl. and *Sesamum indicum* var. *angustifolium* Oliv. *S. calycinum* subsp. *angustifolium* is an erect, annual to perennial herb with or without side branches. It can reach a height of 0.4–2.0 m. The flowers appear pink or purple and often have spots within. Its distribution encompasses eastern tropical Africa, including Uganda, Tanzania, Democratic Republic of Congo, and Kenya and south to Malawi, Zambia, and Mozambique. It is occasionally cultivated as a vegetable and prefers sandy soil. It frequently grows by roadsides, in grasslands, and open woodlands [46]. Local names in different languages in Uganda are as follows: **Luganda:** lutungotungo [14, 21] and **Lusoga:** lutungotungo [21].

#### 3.1.13. *Solanum aculeastrum*

This plant species is a large shrub or small tree, and it was reported to be cultivated in Rugazi, Bynyaruguru, and Ankole in western Uganda [41]. It can reach up to 6 m in height [20]. *S. aculeastrum* is a native African plant that occurs from the South African Cape to the Imatong mountains in Sudan and westwards to Cameroon [47]. Its branchlets are densely covered in woolly hairs and possess sharp, curved thorns [48]. It flowers from September to July, peaking in November and March, and fruits from April to January, peaking in June and November [49]. The fruits are extremely bitter and highly toxic due to the presence of the poisonous alkaloid solanine [24, 50]. The species is regionally known as “bitter apple” [20]. Local names in Uganda are as follows: **Luganda:** ekitengo, entego eddene, and entengo lyabalalo [14, 20, 41]. Local names in other East African countries and languages are as follows: **Kikuyu:** mutura [20], **Kipsigis:** siganet [20], and **Maasai:** osigawai [20].

#### 3.1.14. *Toddalia asiatica*

Synonyms include *Aralia labordei* H.Lév., *Cranzia aculeata* (Sm.) Oken., *Paullinia asiatica* L., *Toddalia aculeata* (Sm.) Pers., and *Toddalia floribunda* Wall. *T. asiatica* is a woody liana or shrub widely distributed in Southeast Asia, South Africa, and tropical Africa [51]. In Uganda, it is cultivated by traditional healers and was designated as a multipurpose slow-growing shrub with important therapeutic values [52]. It commonly grows in tropical forests, especially near anthills, near rivers or streams, and it grows fairly well in clay soils [53]. In East Africa, this indigenous species commonly grows in riverine and forest edge habitats from where it is harvested. Local herbalists in Uganda exclusively harvest it from the wild [54]. Local names in Uganda are as follows: **Luganda:** kawule [14, 55] and **Luo:** ajua [22]. Local names in other East African countries and languages are as follows: **Maasai:** ole-barmonyo [22], **Digo:** chikombe za chui [22], **Kikuyu:** mwikunya [22], **Kamba:** maluia [22], **Luhya:** luabare [22], **Marakwet:** kipkeres [22], **Meru:** mukonguru [22], **Tugen:** ketemwe [22], **Nandi:** usuet [22], **Turkana:** etokebengu [22], and **Samburu:** llaramunyo [22].

#### 3.1.15. *Warburgia ugandensis*

Some common synonyms are *Dawea ugandensis* Sprague ex Dawe and *Warburgia ugandensis* subsp. *ugandensis. W. ugandensis* is an evergreen tree with a dense leafy rounded canopy that is widely distributed in lower rainforest and drier highland forest areas of East Africa. It is also known as the “East African greenheart” and the “pepper-bark tree.” The species can grow up to 25 m high. It occurs between 1,000 and 2,000 m.a.s.l. In Uganda, it grows in colonizing forests, forest edges, and thickets, as well as often on dry sites [20, 22, 23]. *W. ugandensis* is one of the most commonly used multipurpose medicinal plant species in Uganda [14, 21]. It is a fairly slow-growing tree whose seeds quickly lose viability. The wood has high oil content [23]. Local names in Uganda are as follows: **Luganda:** abasi, muya, and mukazanume [14, 20, 21], **Mukuzanume, dialect Buddu:** muwiya [23], **Lusoga:** balwegiira [21, 23], **Lugishu:** balwegira and abasi [21], **Luo (Langi):** abac [21], **Runyoro:** musizambuzi and mwiha [20, 22, 23], **Rutoro:** muharami [20, 22, 23], and **Lugwere:** muwiya [22]. Local names in other East African countries and languages include the following: **Kikuyu:** muthiga [20], **Maasai:** osogonoi and msokonoi [20], **Rangi:** osogonoi and msokonoi [20], **Kisii:** omenyakige [20], **Luhya:** apacha [20], **Meru:** musunui [20], **Nandi:** soget and sorget [20], **Tugen:** soget and sorget [20], **Kipsigis:** sogoet [20], **Goro:** sagonai [20], **Haya:** muhiya [20], and **Sambaa:** mdee and mlifu [20].

#### 3.1.16. *Zanthoxylum chalybeum*

Synonyms include *Zanthoxylum chalybeum* var. *chalybeum* and *Fagara chalybea*. *Z. chalybeum*, also known as the “lemon-scented knobwood,” is a spiny deciduous shrub or tree that can reach up to 8 m in height [20]. Its crown is open rounded. It grows in medium to low altitudes up to 1,500 m.a.s.l., mainly in dry woodlands, bushlands, or grasslands and often on termite mounds and in rocky places. The bole has characteristic large, conical woody knobs with sharp prickles. Twigs and branches have single recurved spines that are up to 2 cm long and dark red. It can be propagated through seeds and cuttings obtained from wild or cultivated plants. The seeds lose viability quickly [20, 23, 30]. The leaves have a strong lemon smell if crushed [30]. Local names in different languages in Uganda are as follows: **Ateso:** eusuk and musuku [20, 23, 30], **Luo (Acholi/Alur):** kichuk and roki [23, 30], **Luganda:** ntaleyedungu, ntaleyaddungu, and ntaliyedongu [14, 21, 23, 30], **Lugwere:** musuku [20, 30], **Lusoga:** ddungu lya ntale [23], **Ik:** rukuts [23], **Lugbara:** outiku [23], **Swahili:** mjafari, mkununungu, and mtata [20, 30], and **So (Tepes):** wangok and ongokat [30]. Local names in other East African countries and languages are as follows: **Maasai:** ol-oisugi and ol oissugu [20, 30], **Zaramo:** mnungu [20], **Nandi:** sagawaita [20], **Kipsigis:** sagawaita [20], **Digo:** mdungu, mdhungu, and mundungu [20, 30], **Chonyi:** mdungu and mdhungu [20], **Giriama:** mdungu and mdhungu [20, 30], **Kamba:** mukenea [20], **Mbeere:** mugucua [20], **Meru:** mugucua [20], **Tharaka:** muguuchwa [20], **Marakwet:** sangoja and songuruwa [30], **Luguru:** mhunungu [20], **Nywarwanda:** intare y'irungu [30], **Teita:** genika [30], **Samburu:** l'oisug-i and l'oisuk-i [30], **Boran:** gàdda [30], **Boni:** arer and arere [30], **Shambaa:** mfuakumbi [30], **Hehe:** mulungulungu [30], **Mbunga:** muhuluhumbi and mulunguhumbi [30], **Nyamwezi:** mnugunugu [30], **Zigua:** mhombo and mkunungu [30], and **Sukuma:** nungunungu [30].

### 3.2. Literature Review

The determination of the DoPs enabled successful assessment of the degrees to which each individual plant species has been studied so far, while also taking into account the methodological “research chain of ethnopharmacology” from ethnobotanical studies (“traditional use”) to pharmacological assays (“bioactivity”) and finally to pharmacognostic research (“structure elucidation”). The significance of a research paper was also assessed by determining whether its journal and publishing house were members of the COPE.

The literature survey was completed on 31 July 2019 and covered the period of 1960–2019. In total, 634 peer-reviewed publications were reviewed, 53.3% of which were published in journals, and by publishing houses affiliated with the COPE (338 publications). These articles were published in 304 different academic journals, of which 114 are COPE members. A cloud-based literature library was successfully created, first categorizing publications according to the selected plant species mentioned in the paper and subsequently, according to their individual DoPs (“Traditional use,” “Bioactivity,” “Structure elucidation,” and “Other”). Excluding the DoP “Other,” there were a total of 441 field-related original research papers, of which 245 were published by journals with COPE membership (55.6%).

### 3.3. DoP Analysis on the Totality of Selected Plant Species

**Figure 2:**
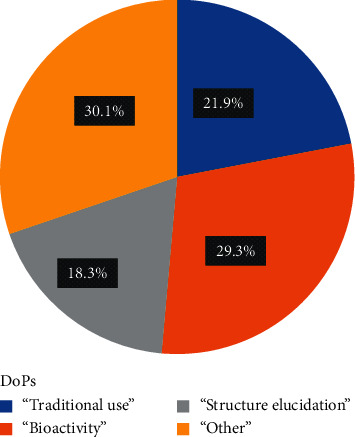
Distribution of peer-reviewed articles within the different DoPs (total number of articles: 634; number of species of interest: 16).

Figure 2 shows the distribution of papers by DoPs. A total of 191 publications (30.1%) were allocated to the DoP “Other,” as these were mostly non-field-related publications and a few review papers. Reference to the plant species of interest was often in the form of documentation of traditional knowledge and medicinal application, and those papers were allocated to the DoP “Traditional use” (139 papers). This represents 21.9% of all original research publications mentioning one of the 16 plant species or about a third of all field-related publications (31.5%). With 186 articles (42.2%), the largest share of field-related publications were classified to the DoP “Bioactivity,” depicting the importance of *in vitro* and *in vivo* evaluation of traditional use and pharmacology activity in the field of ethnopharmacology. DoP “Bioactivity” categorized articles made up 29.3% of all published papers (including DoP “Other”). As this is the final stage of the bioassay-guided fractionation methodology in drug discovery, original research dealing with structure elucidation of bioactive compounds made up the smallest share (DoP “Structure elucidation” = 116), representing 18.3% of all recorded publications and 26.3% of the field-related publications (without DoP “Other”).

### 3.4. Journal Analysis and COPE Assessment

**Figure 3:**
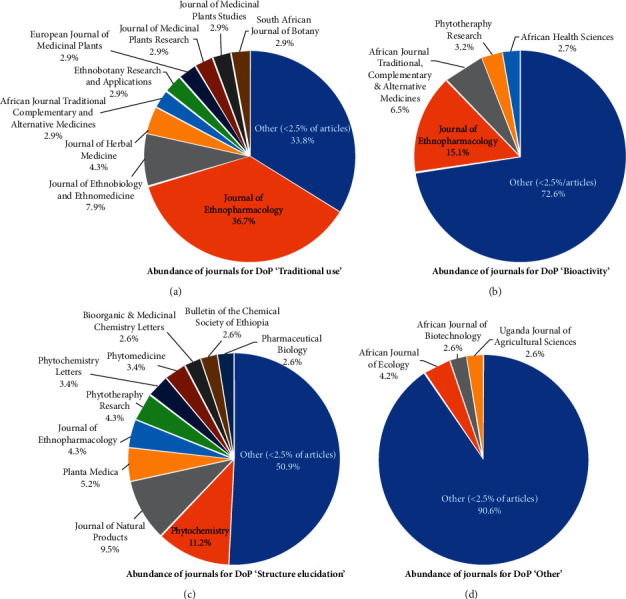
Distribution of scientific articles within peer-reviewed journals (total number of articles: 634; number of species of interest: 16). (a) Abundance of journals for DoP “traditional use.” (b) Abundance of journals for DoP “bioactivity.” (c) Abundance of journals for DoP “structure elucidation.” (d) Abundance of journals for DoP “other.”

Subsequently, the frequency of each DoP term's publication in individual peer-reviewed journals (“abundance of publication”) was assessed. The results are shown in Figure 3. The significance of COPE member *Journal of Ethnopharmacology* (*JEP*) to the field can be affirmed, as by far the greatest proportion of related articles describing “Traditional use” and “Bioactivity” were published in the *JEP*, as well as the fourth-highest number of papers recorded for the DoP “Structure elucidation.” Overall, 36.7 % of all publications categorized under the DoP “Traditional use” (51 articles) were printed in the *JEP*, 15.1% in the case of the DoP “Bioactivity” (28 articles), and 4.3% for the DoP “Structure elucidation” (5 articles).

Other journals that published the greatest proportion of “Traditional use”-related papers on the 16 selected medicinal plants are the *Journal of Ethnobiology and Ethnomedicine* (7.9%, 11 articles, COPE member), the *Journal of Herbal Medicine* (4.3%, 6 articles, COPE member), the *African Journal of Traditional, Complementary and Alternative Medicines* (2.9%, 4 articles), *Ethnobotany Research & Applications* (2.9%, 4 articles), the *European Journal of Medicinal Plants* (2.9%, 4 articles), the *Journal of Medicinal Plants Research* (2.9%, 4 articles), the *Journal of Medicinal Plant Studies* (2.9%, 4 articles), and the *South African Journal of Botany* (2.9%, 4 articles, COPE member). The rest of the “Traditional use”-related publications (33.8%, 47 articles) were printed in 37 other journals, of which the majority fail to be COPE members (26 journals).

In terms of the DoP “Bioactivity,” the second-largest proportion of papers was published in the *African Journal of Traditional, Complementary and Alternative Medicines* (6.5%, 12 articles), followed by *Phytotherapy Research* (3.5%, 6 articles, COPE member) and *African Health Sciences* (2.7%, 5 articles). Ninety-seven other journals with minor article distribution (<2.5%) were identified and summarized under “Other” (72.6%, 135 articles). The majority of these journals are not COPE members (64 journals).

The most dominant journals for the DoP “Structure elucidation” were the COPE members *Phytotherapy* (11.2%, 13 articles) and *Journal of Natural Products* (9.5%, 11 articles). These journals are also followed by COPE members to the biggest part: *Planta Medica* (5.2, 6 articles), the *JEP* (4.3%, 5 articles, COPE member), *Phytotherapy Research* (4.3%, 5 articles, COPE member), *Phytochemistry Letters* (3.4%, 4 articles, COPE member), *Phytomedicine* (3.4%, 4 articles, COPE member), *Bioorganic & Medicinal Chemistry Letters* (2.6%, 3 articles, COPE member), the *Bulletin of the Chemical Society of Ethiopia* (2.6%, 3 articles), and *Pharmaceutical Biology* (2.6%, 3 articles, COPE member). A total of 59 papers were published in 50 other journals (50.9%), of which 24 are COPE members.

Statistical analysis of the DoP “Other” resulted in the identification of three journals that were most abundant: the *African Journal of Ecology* (4.2%, 8 articles, COPE member), the *African Journal of Biotechnology* (2.6%, 5 articles), and the *Uganda Journal of Agricultural Sciences* (2.6%, 5 articles). Moreover, the majority of papers, including their corresponding journals, were summarized as “Other” (<2.5% of articles in DoP “Other” published in this journal), consisting of a total of 173 articles (90.6%) printed in 138 different journals, whereas only 58 of these are COPE members. Research categorized under the DoP “Other” and its individual journals was diverse, ranging from journals on botany (e.g., *Planta*, *Systematic Botany*, and *American Fern Journal*), nature conservation (e.g., *Biological Conservation* and *Journal of Threatened Taxa*), geography (e.g., *Applied Geography* and *Journal of Biogeography*), ecology (e.g., *Journal of Chemical Ecology*, *Plant Ecology*, *Oecologia*, and *Advanced Journal of Ecology and Ecosystems*), animal sciences (e.g., *Journal of Advanced Veterinary and Animal Research* and *Livestock Science*), and insect studies (e.g., *Journal of Applied Entomology*, *Journal of Insect Physiology*, *Applied Entomology and Zoology*, and *Entomologia Experimentalis et Applicata*) to more abstract journals (e.g., *Polymers*, *International Journal of Creative Research Thoughts*, *International Journal of Cosmetic Science*, *Journal of Archaeological Science*, and *Digest Journal of Nanomaterials and Biostructures, Biomass and Bioenergy*), and others.

### 3.5. Assessment of Study Progress for Each Species

**Figure 4:**
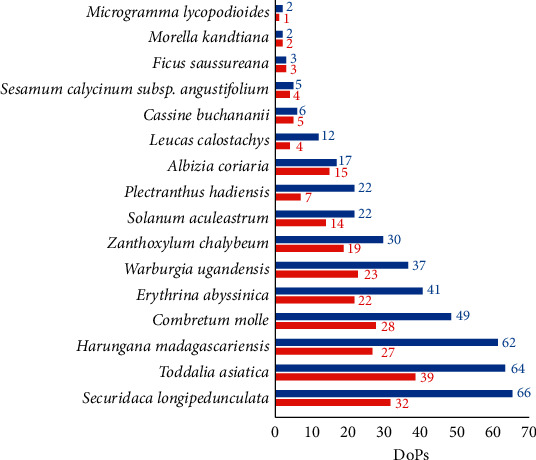
Summarized “total” DoPs (without “Other”) for assessment of degree to which the species have been studied so far; blue = total number of journal articles; red = number of journal articles from publishing houses with COPE membership.

The DoPs were used as a tool for assessment of the degree to which a species has been studied so far. Results on individual plants are shown in Figure 4 (accumulated DoPs, excluding the DoP “Other”) and Table 2 (absolute values of individual DoP categories). Values in red or in square brackets state the total number of articles published in journals with COPE membership that committed themselves to reaching highest standards and best practice in scholarly publication ethics.

#### 3.5.1. Highly Understudied Species

Plant species identified as being highly understudied are *M. lycopodioides* (DoP_total_ = 2 (1)), *M. kandtiana* (DoP_total_ = 2 (2)), *F. saussureana* (DoP_total_ = 3 (3)), *S. calycinum* subsp. *angustifolium* (DoP_total_ = 5 (4)), *C. buchananii* (DoP_total_ = 6 (5)), and *L. calostachys* (DoP_total_ = 12 (4)). Numbers in brackets correspond to absolute numbers of journal articles from publishing houses with COPE membership.

According to results of our literature review, *M. lycopodioides* has been studied and mentioned in a total of 63 journal articles over the past 60 years. However, the vast majority of these papers deal with non-medicinal use and are not part of drug discovery-related research. The papers mainly describe the occurrence of the fern species in the American tropical forests and its extraordinary morphology/biology; traditional medicinal use has only been mentioned in two publications so far. Here, it has been reported to be used for removal of lice and to treat anemia in South Africa and Tanzania [56]. The second publication reports traditional use by the Zambo-Miskito ethnic group of Eastern Nicaragua to cure bewitchment and chase away evil spirits [57].

Traditional use of the shrub *M. kandtiana* has also only been previously described in two publications following ethnobotanical surveys. Just as in our ethnobotanical study in the Greater Mpigi region [14], its roots were cited to be used in treatment of HIV/AIDS in one of the four surveyed Ugandan districts [58]. The second paper names *M. kandtiana* bark, leaves, and fruits as a natural remedy in treatment of tuberculosis (fruits, leaves, roots, and bark used) in the Butambala and Mpigi Districts, which constitute a major part of the Greater Mpigi region [43]. In our ethnobotanical survey [14], we were able to confirm these citations of traditional use in the study area (root bark, leaves, and roots). There were no other publications from any other field identified, mentioning *M. kandtiana*.

As far as *M. lycopodioides* and *M. kandtiana* are concerned, no studies on bioactivity or isolation and elucidation of active secondary metabolites have been published so far.

In the case of *L. calostachys,* research has shifted slightly towards the investigation of pharmacological activity. The majority of papers mention its traditional medicinal use (DoP_traditional use_ = 9 (3)), while three publications investigated the antiplasmodial activity of the plant (DoP_bioactivity_ = 3 (1)), reporting moderate to low antiplasmodial activity of crude extracts [59–61]. Traditional uses were recorded in Kenya only and encompass treatment of malaria [62, 63], gastrointestinal disorders [64–68], ulcers [65, 67], tructure elucidation of compounds from P. pneumonia [69], colic pain in infants [65], stomach ache [68, 69], heartburn [65, 67], cough [70], amoebiasis [65], headache [65], heart diseases [65], renal disorders [65], flu [68, 70], arthritis [65], skin diseases [65], and cancer [65]. There were no articles found describing isolation and identification of bioactive natural products from *L. calostachys*.

*F. saussureana, S. calycinum* subsp. *angustifolium*, and *C. buchananii* are still highly understudied, but research has progressed to the bioanalytical stage of structure identification.

Regarding *F. saussureana*, the intra- and interspecific variations in vacuolar flavonoids among *Ficus* species from the Budongo Forest, Uganda, were described [71]. Another paper, which undertook a pharmacological evaluation of bioactivity, investigated the vasodilating effect of the root bark extract of *F. saussureana* on the guinea pig aorta [72]. In addition, *F. saussureana* has also recently been mentioned in Ugandan traditional medicine for the use of its leaves and stem bark in the treatment of HIV/AIDS in parts of the Greater Mpigi region [17].

Only four publications mentioned the traditional use of *S. calycinum* subsp. *angustifolium*; however, there was one article on bioactive natural product structures isolated from this species. The first paper classifies *S. calycinum* subsp. *angustifolium* as a weed that is used medicinally as an emetic and contraceptive, as well as for treatment of eye diseases, diarrhea, burns, and wounds by the Haya people in the Kagera region, Tanzania [73]. In the second paper, Kibuuka and Anywar describe the traditional use of *S. calycinum* subsp. *angustifolium* against hernias in Central Uganda [16]. The third paper mentions the use of the fresh leaves in treatment of hypertension in Bulamogi county, Uganda (eaten together with *Arachis hypogaea* or *Sesamum indicum*) [74]. The fourth ethnobotanical study from the DoP_traditional use_ was conducted in rural and urban areas across Central Uganda (including part of the Greater Mpigi region). The article mentions the use of the root powder (1 tablespoon) in 200 mL water, which is boiled for 5 minutes and then drunk twice a day to induce vomiting [75]. In terms of the DoP_structure elucidation_, Chidewe et al. isolated six compounds from *S. calycinum* subsp. *angustifolium* [76]. Two were already known (the hydrocarbon nonacosane and the glucosinolate glucoiberverin), while the other four remained unidentified and were not structure elucidated.

*C. buchananii* was mentioned twice as being used in other African countries and cultures, namely, as a natural remedy against fungal infections in the southern highlands of Tanzania [77] and against back pain, hernia, and erectile disfunction in the Kagera region, northwest Tanzania [78]. At our study site in the Greater Mpigi region [14], we also documented the common practice of using this plant medicinally in the treatment of erectile dysfunction. The Kagera region is relatively close to the Greater Mpigi region and also situated in the Lake Victoria basin. Among other Tanzanian medicinal plants traditionally used to treat fungal infections, *C. buchananii* has been investigated for cytotoxic, genotoxic, and CYP450 enzymatic competition effects [79]. In addition, in the 1990s, three compounds were isolated from *C. buchananii*: (1) elabunin, a novel dammarane triterpene from the root bark with moderate cytotoxic activity against L-1210 leukemic cells [80]; (2) mutangin, a novel sesquiterpene from the plant's fruit, with moderate antifeedant activity [81]; and (3) buchaninoside, a steroidal glycoside from the plant's fruit, with antifeedant activity against *Spodoptera exempta* larvae [82].

#### 3.5.2. Understudied Species

Plant species identified as still being understudied are *A. coriaria* (DoP_total_ = 17 (15)), *P. hadiensis* (DoP_total_ = 22 (7)), and *S. aculeastrum* (DoP_total_ = 22 (14)).

In contrast to *M. lycopodioides*, *M. kandtiana*, and *L. calostachys*, studies on *A. coriaria* (DoP_structure_elucidation_ = 2 (2))*, P. hadiensis* (DoP_structure_elucidation_ = 6 (3)), and *S. aculeastrum* (DoP_structure_elucidation_ = 6 (2)) already resulted in isolation of secondary metabolites and structure elucidation [83–96].

*A. coriaria* was reported to be used traditionally in 11 publications that show results of surveys conducted in Uganda, except for the one in Kenya. Forty traditional healers from the Greater Mpigi region named *A. coriaria* as one of their priority plants for the treatment of tuberculosis [43]. Two more studies from Mpigi district identified the stem bark and the leaves for treatment of HIV/AIDS and related medical disorders [17, 58]. At another study site in Uganda, Katabi subcounty in Wakiso district, the leaves and the bark were used in treatment of wounds and skin rashes [97]. In Kakamega county, Kenya, the bark and the leaves were reported to be used in treatment of breast, uterine, and skin cancer [98]. Another publication assessed the ecological status and ethnobotany of *A. coriaria* in Budondo subcounty, eastern Uganda, highlighting its use, local harvesting patterns, and local attitudes towards its conservation. Community members consider the species as being rare, and abundance is declining in the region. Among many non-medicinal uses, the root and the stem bark were used in the treatment of syphilis, skin diseases, jaundice, eye diseases, cough, sore throat, and to concentrate breast milk in humans [99]. Another study assessed the possibility of setting up multipurpose tree gardens to provide traditional healers with species used for medicine [100]. In the county of Bulamogi in Uganda, the bark is used by traditional healers in the treatment of diarrhea, “lameness” (*butenge*), syphilis, snake bites, and amoebiasis. The roots are used for the treatment of pyomyositis and amoebiasis. The leaves were also reported to be used against snake bites [74]. In Kibale rainforest, an aqueous decoction of the fresh stem bark is drunk to treat cough [101]. One Ugandan study assessed the domestication of medicinal tree species in the Victoria lakeshore region, including *A. coriaria*, and their distribution by vendors on local markets [102]. With regard to the DoP_bioactivity_, four publications were recorded. One article reported low antigiardial activity of a mixture of roots and bark against *Giardia lambia* at 500 *µ*g/mL [64]. Another study investigated the stem bark and reported moderate *in vitro* antiplasmodial activity against *Plasmodium falciparum* D6 (IC_50_: 37.83 *µ*g/mL) and low antileishmanial activity [103]. A dichloromethane extract of the stem bark displayed moderate antiplasmodial activity against *P. falciparum* D6 (IC_50_: 10.68 *µ*g/mL) and the chloroquine-resistant *P. falciparum* W2 strain (IC_50_: 6.80 *µ*g/mL) [104]. Three stem bark extracts with varying solvents displayed moderate to low growth inhibitory activity against five African livestock pathogens of the genus *Mycoplasma* [105]. Publications categorized under the DoP_structure elucidation_ include reporting of the isolation of two new oleanane-type saponins, coriariosides A and B, along with a known saponin, gummiferaoside C, from the roots of *A. coriaria*. As part of this study, coriarioside A and gummiferaoside C displayed cytotoxic activity against the colorectal human HCT116 and HT29 cancer cells [83]. The same group of researchers published the isolation and structure elucidation of coriarioside C, D, and E from the roots shortly after [84].

*P. hadiensis* has been only mentioned to be traditionally used (DoP_traditional use_) in three ethnobotanical studies so far. One paper described the use of leaves for wound healing in the Malabar Region of Kerala, India [106]. Another article reported the use of seeds and stem bark as a fishing poison in South Africa [107]. The third article mentioned *P. hadiensis* and related species in the context of medicinal plant use to treat respiratory infections, digestive disorders, and skin infections [108]. Following these ethnobotanical data, the plant's leaves have recently been reported in a preliminary study to display antibacterial activity against *S. aureus*, isolated from wounds in Bushenyi district, Uganda [109]. A study investigated the antibacterial effect of the essential oil from the aerial parts against *P. aeruginosa*, *S. aureus*, *E. coli*, and *S. mutans*, which showed no significant activity [110]. Another study published by the same group investigated the antioxidant activity of the aerial parts [111]. A leaf extract displayed low larvicidal activity against the fourth instar larvae of *Aedes aegypti*, a dengue fever vector (LC_50_: 489.278 *µ*g/mL) [112]. A terpene-rich methanolic extract of the shoot part and a methanolic extract of the stem was investigated for cytotoxicity using a shrimp brine lethality assay (LC_50_: 145 *µ*g/mL) and against HeLa cells (141.3 *μ*g/mL) [113, 114]. Other studies include (a) antibacterial susceptibility single dose and antioxidant activity studies [115–117]; (b) two studies investigating the antioxidant, antiproliferative, and antiinflammatory properties of the shoot parts [118, 119]; and (c) a study screening several *Plectranthus* species, including *P. hadiensis*, for antiinflammatory effects [120]. Isolation and structure elucidation of compounds from *P. hadiensis* (DoP_structure__elucidation_) resulted in five new abietane-type diterpenoids (7*β*-acetoxy-6*β*-hydroxyroyleanone, 7*β*, 6*β*-dihydroxyroyleanone, 11,20-dihydroxysugiol, 11-hydroxysugiol, 1,11-epoxy-6,12-dihydroxy-20-norabieta-1(10), and 5,8,11,13-pentaen-7-one) [91, 92], a known stereoisomer (7*α*-acetoxy-6*β*-hydroxyroyleanone) [91], carnosolon [92], and 25 known compounds detected in the essential oil extracted from the seeds [95]. A terpenoid fraction of *P. hadiensis*, containing 1-octern-ol, linalool, nerol, Z-citral, geraniol, neryl acetate, *α*-copaene, geranyl acetate, *δ*-cadinene, *β*-cubebene, *α*-cadinol, and valencene induced apoptosis in human colon cancer HCT15 cells [96].

Concerning *S. aculeastrum* and the DoP_traditional use_, multiple medicinal uses were recorded. These include traditional use of (a) the berries and leaves in treatment of lymphatic filariasis in the KwaZulu-Natal and Mpumalanga regions of South Africa [121]; (b) the berries, leaves, roots, and bark against cancer in the Kakamega county of Kenya and Eastern Cape Province of South Africa [98, 122]; (c) the roots to treat stomachache in Limpopo Province of South Africa [123]; and (d) the berry juice against *ditlapedi* (a facial skin condition) in the Central Sekhukhuneland of South Africa [124]. Although the practice is regarded by Rwandan women as “a positive force in their lives,” *S. aculeastrum* has been described as being used as medicine applied during stretching sessions for elongation of the labia minora, which is classified as Type IV female genital mutilation by the World Health Organization [125]. Publications, categorized under the DoP_bioactivity_, reported low antioxidant activity of the berries and low antimicrobial activity of the berries and leaves against ten bacterial and five fungal strains [126–129]. A methanolic extract from the berries displayed low activity in inhibiting the growth of promastigotes in *Leishmania major* infection in BALB/c mice (IC_50_: 78.62 *μ*g/ml) [130]. Methanolic extracts of the berries showed antiproliferative activity against human HeLa, MCF7, and HT29 tumour cell lines, while the leaf extracts displayed no cytotoxic activity [131]. In another study, the methanolic extracts of the leaves and the berries showed moderate activity against host snails of schistosomiasis [132]. MeOH-CH2Cl2 (1 : 1, v/v) extracts from the stem bark and the berries showed growth inhibitory activity against five African livestock pathogens of the genus *Mycoplasma*, displaying a mean MIC value of 20 *µ*g/mL [105]. One acute toxicity study of an extract of the unripe berries in Wistar rats resulted in toxicity symptoms such as respiratory distress, epistaxis, and hypoactivity that disappeared 72 h after treatment. Above 125 mg/kg body weight, the extract produced mortality in the Wistar rats, and the latency was inversely proportional to the doses [133]. Another study investigated the toxicological effect of the aqueous extract of fresh, dried, and boiled berries in male Wistar rats at 1, 10, and 25 mg/kg body weight for 28 days. The rats gained weight, but showed no signs of clinical toxicity at the doses tested [134]. Concerning the DoP_structure elucidation_, two new steroidal alkaloids were isolated from the root bark, along with known compounds such as solamargine and *ß*-solamargine [89, 90]. Steroid alkaloids, namely, solasodine and tomatidine, isolated from the berries, displayed cytotoxic effects on the growth of HeLa, MCF7, and HT29 cancer cell lines and antioxidant properties [87, 88]. Solamargine, isolated from the berries, induced nonselective cytotoxicity and P-glycoprotein inhibition [85]. Volatile oil fractions from the leaves and berries were investigated via GC-MS analysis and contained mainly alkanes and alkenes [86].

#### 3.5.3. Moderately Studied Species

*Z. chalybeum* (DoP_total_ = 30 (19)), *W. ugandensis* (DoP_total_ = 37 (23)), and *E. abyssinica* (DoP_total_ = 41 (22)) were classified as having been moderately studied in the past. A discussion of all the published literature was not conducted at this point because this would merit its own standalone review article for each of the three species. Review articles of the genera *Warburgia* [135, 136], *Zanthoxylum* [137], and *Erythrina* [138] have been published.

#### 3.5.4. Highly Studied Species

Plant species identified as having been highly studied in the past are *C. molle* (DoP_total_ = 49 (28)), *H. madagascariensis* (DoP_total_ = 62 (27)), *T. asiatica* (DoP_total_ = 64 (39)), and *S. longipedunculata* (DoP_total_ = 66 (32)). A discussion of all the published literature was not conducted at this point because this would merit its own standalone review article for each of the four species. A review article of the genus *Combretum* [139], a minireview article of *H. madagascariensis* [140], and a review article of *S. longipedunculata* [141] have been published.

## 4. Conclusions

An extensive literature survey successfully assessed the degree to which a plant species has been studied so far, introducing a new indicator: the Degrees of Publication (DoPs). This literature assessment resulted in the identification of understudied plants among the selected 16 species. Three plant species were identified as being moderately studied (*E. abyssinica*, *W. ugandensis*, and *Z. chalybeum*), while four have already been highly studied over the past decades (*C. molle*, *H. madagascariensis*, *S. longipedunculata*, and *T. asiatica*). More importantly, the majority of plant species surveyed have not yet been investigated sufficiently. Six species were classified as being highly understudied (*C. buchananii*, *F. saussureana*, *L. calostachys*, *M. lycopodioides*, *M. kandtiana*, and *S. calycinum* subsp. *angustifolium*) and three more species as being understudied (*A. coriaria*, *P. hadiensis*, and *S. aculeastrum*). Due to the absence of any bioactivity-related publications for *S. calycinum* subsp. *angustifolium*, *M. lycopodioides*, and *M. kandtiana*, pharmacological evaluation of these species should be prioritized. The need for research and development of novel natural products is more vital than ever, making future studies on traditional herbal remedies justified and urgently required.

Generally, there was no significant correlation between the DoPs (Figure 4) and the RFCs (Table 1) of individual plant species, previously published by Schultz et al. [14]. This is most likely due to the fact that the species are highly used within the study area (Greater Mpigi region, Uganda) but occur interregionally on the African continent, and some are even native to other continents (e.g., *M. lycopodioides* or *T. asiatica*). From the literature survey, it was noted that other natural plant habitats and ethnic groups than the people of the Greater Mpigi region were more often surveyed in the past, followed by ethnopharmacological lab studies of some of the plant species. In conclusion, the discrepancy between high regional medicinal use and low DoPs confirms the demand for studying the diverse cultures and ethnomedical practices of the Greater Mpigi region, in particular, and the 16 selected species, in general.

The new DoPs indicator proved to be a valuable tool that fills a gap compared to other ethnopharmacological tools. Other than existing field assessment tools, e.g., the relative frequency of citation, the fidelity level, the use value, and the informant consensus factor, the DoPs tool can be leveraged to better identify those species that are understudied and merit deeper investigation. This includes using the tool for selection of species for costly lab studies, thereby avoiding reproduction of results, while facilitating a more time-efficient approach to ethnopharmacological research. When applied by other researchers in the future, another value of the new tool will be that gaps in the literature could be filled more strategically, making ethnopharmacological research more targeted and efficient.

It should be noted that focusing on a logic approach to the field of ethnopharmacology, such as (a) starting with ethnobotanical/ethnopharmacological field studies; (b) continuing with validation of traditional medicine via *in vitro* and *in vivo* pharmacological assays in the lab, and (c) progressing to bioassay-guided fractionation, natural product isolation and structure elucidation studies, cannot always, though in most cases, be applied within the field. One such example would be ritual use or where pharmacological effects cannot be “measured,” which would still be of value to the field, e.g., with symbolic significance to an indigenous community. Here, it would be categorized under the DoP_traditional use_, thus recognized, and lab studies may not follow.

## Figures and Tables

**Table 1:** Overview of medicinal plant species investigated in this study, indicating high traditional use in treatment of medical disorders in the Greater Mpigi region (*n* = 39).

Botanical name	Local name (Luganda language)	Family	RFC (%)
*Albizia coriaria* Oliv.	Mugavu	Fabaceae	100.0
*Cassine buchananii* Loes.	Mbaluka	Celastraceae	61.5
*Combretum molle* R.Br. ex G.Don	Ndagi	Combretaceae	89.7
*Erythrina abyssinica* DC.	Jjirikiti	Fabaceae	100.0
*Ficus saussureana* DC.	Muwo	Moraceae	94.9
*Harungana madagascariensis* Lam. ex Poir.	Mukabiiransiko	Hypericaceae	97.4
*Leucas calostachys* Oliv.	Kakuba musulo	Lamiaceae	43.6
*Microgramma lycopodioides* (L.) Copel.	Kukumba	Polypodiaceae	43.6
*Morella kandtiana* (Engl.) Verdc. & Polhill	Mukikimbo	Myricaceae	87.2
*Plectranthus hadiensis* (Forssk.) Schweinf. ex Sprenger	Kibwankulata	Lamiaceae	97.4
*Securidaca longipedunculata* Fresen.	Mukondwe	Polygalaceae	38.5
*Sesamum calycinum* subsp. *angustifolium* (Oliv.) Ihlenf. & Seidenst.	Lutungotungo	Pedaliaceae	87.2
*Solanum aculeastrum* Dunal	Kitengo	Solanaceae	71.8
*Toddalia asiatica* (L.) Lam.	Kawule	Rutaceae	97.4
*Warburgia ugandensis* Sprague	Abasi	Canellaceae	92.3
*Zanthoxylum chalybeum* Engl.	Ntaleyaddungu	Rutaceae	46.2

**Table 2:** Literature survey overview: DoPs and individual categories; number = total number of journal articles published for a certain DoP category, (COPE) = number of journal articles from publishing houses with COPE membership.

Plant species	Degree of publication (DOP)				
	Traditional use	Bioactivity	Structure elucidation	Other	Total (without “other”)
	Number (COPE)	Number (COPE)	Number (COPE)	Number (COPE)	Number (COPE)
*Albizia coriaria*	11 (9)	4 (4)	2 (2)	9 (5)	17 (15)
*Cassine buchananii*	2 (1)	1 (1)	3 (3)	1 (1)	6 (5)
*Combretum molle*	8 (7)	30 (15)	11 (6)	21 (8)	49 (28)
*Erythrina abyssinica*	18 (9)	7 (2)	16 (11)	7 (4)	41 (22)
*Ficus saussureana*	1 (1)	1 (1)	1 (1)	2 (2)	3 (3)
*Harungana madagascariensis*	11 (5)	38 (12)	13 (10)	11 (2)	62 (27)
*Leucas calostachys*	9 (3)	3 (1)	—	1 (-)	12 (4)
*Microgramma lycopodioides*	2 (1)	—	—	61 (25)	2 (1)
*Morella kandtiana*	2 (2)	—	—	—	2 (2)
*Plectranthus hadiensis*	3 (2)	13 (2)	6 (3)	24 (13)	22 (7)
*Securidaca longipedunculata*	28 (16)	26 (8)	12 (8)	13 (1)	66 (32)
*Sesamum calycinum* subsp. *angustifolium*	4 (3)	—	1 (1)	9 (8)	5 (4)
*Solanum aculeastrum*	6 (5)	10 (7)	6 (2)	3 (2)	22 (14)
*Toddalia asiatica*	15 (6)	21 (12)	28 (21)	9 (8)	64 (39)
*Warburgia ugandensis*	8 (7)	15 (5)	14 (11)	16 (11)	37 (23)
*Zanthoxylum chalybeum*	11 (9)	17 (8)	2 (2)	6 (3)	30 (19)

## Data Availability

The data supporting this bibliographic assessment are from previously published studies, which have been cited. Individual information on publications categorized under different Degrees of Publication is available from the corresponding author upon request.
